# P-2303. Active Surveillance and Antimicrobial Prophylaxis to Prevent Hyperammonemia Syndrome in Lung Transplant Recipients: A Single Center Cohort Study

**DOI:** 10.1093/ofid/ofae631.2456

**Published:** 2025-01-29

**Authors:** Drew Charles, Sara Lee, Ananth Charya, Michael Eberlein, Kapil K Saharia

**Affiliations:** Medical University of South Carolina , Charleston, South Carolina; University of Maryland Medical Center, Baltimore, Maryland; University of Maryland School of Medicine, Baltimore, Maryland; University of Maryland School of Medicine, Baltimore, Maryland; University of Maryland School of Medicine, Baltimore, Maryland

## Abstract

**Background:**

Hyperammonemia syndrome (HS) is a life-threatening complication primarily affecting lung transplant recipients (LTR) and is associated with *M. hominis* and/or *Ureaplasma* spp infection. We implemented an active surveillance and prophylaxis protocol to improve outcomes associated with HS in LTR. Herein, we evaluate the impact of our protocol on outcomes of HS.

**Figure:**
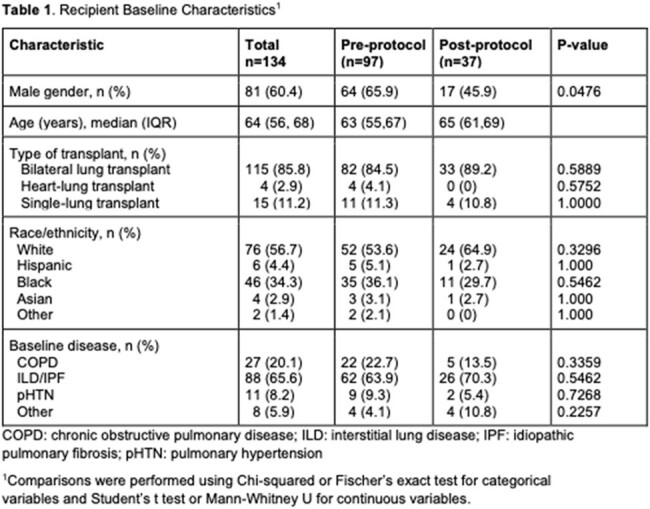

**Methods:**

This is a single-center retrospective cohort study of adult LTR performed from 1/1/2019 to 1/31/2024. Our surveillance and prophylaxis protocol was implemented on 8/1/2022 and included PCR testing for *M. hominis* and *Ureaplasma spp* on post-operative bronchoscopy samples, measurement of serum ammonia levels, and azithromycin prophylaxis in our LTR. Comparisons between groups were performed using Chi-square or Fischer’s exact test for categorical variables and Student’s t test or Mann-Whitney U for continuous variables.

**Figure:**
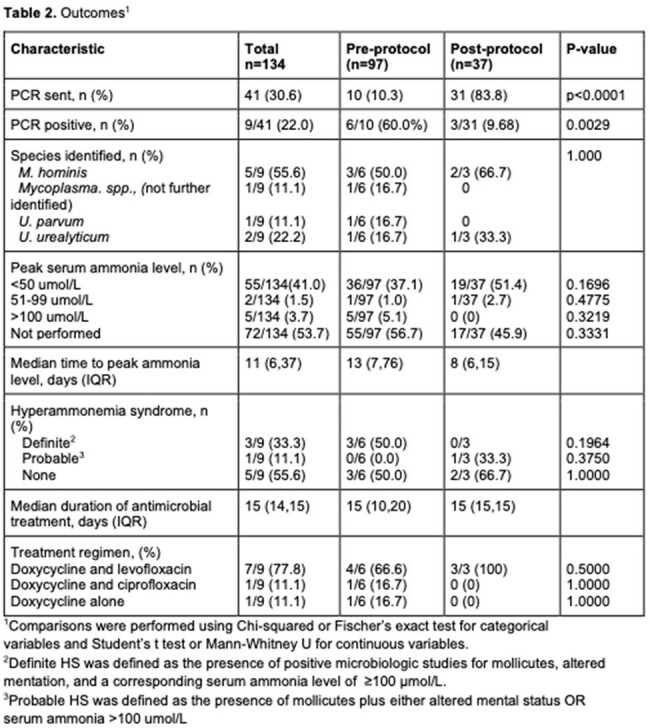

**Results:**

One hundred and thirty-four adults received a lung transplant during the study period. Baseline characteristics are shown in Table 1. In total, 9 LTR (6.7%) were diagnosed with *M. hominis* or *Ureaplasma spp.* infection (Table 2). Of these, 3 LTR met criteria for definite HS (3/134, 2.2%) of whom two died. An additional 3 LTR had disseminated infection due to *M. hominis* without evidence of HS.

Following protocol implementation, 31/37 (83.8%) LTR had PCR testing for *M. hominis* and *Ureaplasma spp.* (Table 2). Ammonia levels were sent within 3 days of transplantation in 20/37 (54.1%) LTR. All patients received prophylaxis with azithromycin (or alternative agent) for at least 5 days or until PCR returned negative. Three LTR had either *M. hominis* or *Ureaplasma spp* infection detected following protocol implementation; none developed HS. One LTR developed disseminated *M. hominis* infection. However, screening was delayed by 10 days and prophylaxis was stopped after 5 days, prior to screening results being known.

**Conclusion:**

The prevalence of HS in our cohort is similar to published reports. Adherence to our protocol was high for azithromycin prophylaxis and PCR testing, but low for ammonia levels. Importantly, no case of definite HS occurred following protocol implementation. Additional studies to determine optimal screening and prophylaxis strategies for HS are needed.

**Disclosures:**

Kapil K. Saharia, MD, MPH, Eurofins Viracor: Advisor/Consultant

